# Obesity is a risk factor for developing critical condition in COVID‐19 patients: A systematic review and meta‐analysis

**DOI:** 10.1111/obr.13095

**Published:** 2020-07-19

**Authors:** Mária Földi, Nelli Farkas, Szabolcs Kiss, Noémi Zádori, Szilárd Váncsa, Lajos Szakó, Fanni Dembrovszky, Margit Solymár, Eszter Bartalis, Zsolt Szakács, Petra Hartmann, Gabriella Pár, Bálint Erőss, Zsolt Molnár, Péter Hegyi, Andrea Szentesi, KETLAK Study Group

**Affiliations:** ^1^ Institute for Translational Medicine, Medical School University of Pécs Pécs Hungary; ^2^ Szentágothai Research Centre University of Pécs Pécs Hungary; ^3^ Doctoral School of Clinical Medicine University of Szeged Szeged Hungary; ^4^ Institute of Bioanalysis Medical School, University of Pécs Pécs Hungary; ^5^ Faculty of Medicine University of Medicine, Pharmacy, Science and Technology of Targu Mures Targu Mures Romania; ^6^ Institute of Surgical Research University of Szeged Szeged Hungary; ^7^ Division of Gastroenterology, First Department of Medicine, Medical School University of Pécs Pécs Hungary; ^8^ Department of Anesthesiology and Intensive Therapy Poznan University for Medical Sciences Poznan Poland; ^9^ First Department of Medicine University of Szeged Szeged Hungary

**Keywords:** COVID‐19, intensive care, mechanical ventilation, obesity

## Abstract

The disease course of COVID‐19 varies from asymptomatic infection to critical condition leading to mortality. Identification of prognostic factors is important for prevention and early treatment. We aimed to examine whether obesity is a risk factor for the critical condition in COVID‐19 patients by performing a meta‐analysis. The review protocol was registered onto PROSPERO (CRD42020185980). A systematic search was performed in five scientific databases between 1 January and 11 May 2020. After selection, 24 retrospective cohort studies were included in the qualitative and quantitative analyses. We calculated pooled odds ratios (OR) with 95% confidence intervals (CIs) in meta‐analysis. Obesity was a significant risk factor for intensive care unit (ICU) admission in a homogenous dataset (OR = 1.21, CI: 1.002‐1.46; I2 = 0.0%) as well as for invasive mechanical ventilation (IMV) (OR = 2.05, CI: 1.16‐3.64; I2 = 34.86%) in COVID‐19. Comparing body mass index (BMI) classes with each other, we found that a higher BMI always carries a higher risk. Obesity may serve as a clinical predictor for adverse outcomes; therefore, the inclusion of BMI in prognostic scores and improvement of guidelines for the intensive care of patients with elevated BMI are highly recommended.

**Abbreviations**
ARDS: acute respiratory distress syndrome
BMI: body mass index
COVID‐19: Coronavirus Disease‐19
ICU: intensive care unit
IL: interleukin
IMV: invasive mechanical ventilation
MCP: monocyte chemoattractant protein
OR: Odds ratio
PMG: Cochrane Prognosis Methods Group
PRISMA: Preferred Reporting in Systematic Reviews and Meta‐analyses
QUIPS: Quality in Prognostic Studies
SARS‐CoV‐2: Severe Acute Respiratory Syndrome Coronavirus 2
TNF: tumour necrosis factor
WHO: World Health Organization
CI: confidence interval
RAS: renin‐angiotensin system,

## 1 INTRODUCTION

In December 2019, a series of pneumonia cases of unknown origin emerged in Wuhan, China. On 7 January 2020, a novel coronavirus, Severe Acute Respiratory Syndrome Coronavirus 2 (SARS‐CoV‐2) was identified as the source of infection.^1^ As of 14 June 2020, Coronavirus Disease‐19 (COVID‐19) infection has been reported in more than seven million cases.^2^

The disease course of COVID‐19 varies in a broad spectrum, from asymptomatic infection to influenza‐like symptoms as well as to severe pneumonia leading to acute respiratory distress syndrome (ARDS).^2^ Recognition of prognostic factors associated with adverse clinical outcomes is essential for prevention and early treatment. Certain diseases, including diabetes mellitus, hypertension, cardiovascular diseases or different types of cancer, have been already identified as predisposing factors to adverse outcomes in COVID‐19.^3^ As these diseases are frequently associated with excessive body fat mass resulting in various hormonal, metabolic and inflammatory changes, adipose tissue may play an important role in the mechanism of the progression of COVID‐19 and obesity might be an important risk factor as well.^4^, ^5^, ^6^, ^7^ Patients with obesity constantly have higher leptin and lower adiponectin levels, as well as they have higher concentrations of pro‐inflammatory cytokines such as tumour necrosis factor (TNF)‐alpha, monocyte chemoattractant protein (MCP)‐1 and interleukin (IL)‐6 produced mainly by adipose tissue, which may contribute to the impaired immune response.^8^, ^9^ These conditions may influence inflammatory and immune responses. Another contributor could be the sedentary lifestyle alone or together with insulin resistance that influences the immune response to microbial agents by impaired macrophage differentiation and modulation of proinflammatory cytokine levels giving way to the invasion of infectious pathogens.^10^, ^11^

Interestingly, the obesity survival paradox has been described in certain diseases such as community‐acquired pneumonia, where despite the increased risk of developing pneumonia, an inverse association could be observed between obesity and mortality.^6^ To the contrary, in the 2009 H1N1 pandemic, obesity was identified as an independent risk factor of severe disease, hospitalization and death.^5^, ^12^, ^13^ Recent data have suggested that obesity, as seen in H1N1, can also be a disadvantageous factor to COVID‐19.^14^, ^15^

Although several articles discussing the impact of the body mass index (BMI) on the need for intensive care unit (ICU) admission or invasive mechanical ventilation (IMV) in COVID‐19 are available, a definite conclusion has not been drawn yet. Therefore, we aimed to clarify the association between the patients' BMI and ICU admission and IMV requirement.

## 2 METHODS

We report our systematic review and meta‐analysis in accordance with the Preferred Reporting Items for Systematic Reviews and Meta‐Analyses (PRISMA) Statement (Table S1).^16^ The review protocol was registered on the PROSPERO International Prospective Register of Systematic Reviews (CRD42020185980). We did not deviate from the protocol except for expanding the exclusion criteria. We decided to perform additional meta‐regressions to assess the correlation between BMI values and rate of ICU admission/IMV. These analyses had their own additional eligibility criteria (detailed in Section 2.2.).

### 2.1 Search strategy

A systematic search was performed in five scientific databases—Medline (via PubMed), Embase, Cochrane Central Register of Controlled Trials (CENTRAL), Scopus and Web of Science—for studies published between 1 January 2020 and 11 May 2020. The following search key was used in all databases without any other filters or restrictions: (‘covid 19') OR (‘Wuhan virus') OR (‘coronavirus') OR (‘2019 nCoV') OR (‘SARS‐cov‐2'). Reference lists of the eligible articles and the citing articles (via Google Scholar search engine) were also screened to capture all relevant studies.

### 2.2 Selection and eligibility criteria

After the removal of duplicates using a reference management software (EndNote X9, Clarivate Analytics), three pairs of review authors independently screened titles, abstracts and then full‐texts against predefined eligibility criteria (each pair screened one‐third of the pool of records) to accelerate the selection process. A third review author resolved the conflicts.

There were no restrictions on the study designs eligible for inclusion. The inclusion criteria specified any peer‐reviewed studies that reported on BMI classes of patients with confirmed SARS‐CoV‐2 infection comparing the proportion of ICU admission or IMV requirement between each group. We excluded studies with fewer than 10 patients.

Regarding meta‐regression, those studies were eligible that contained information on mean/median BMI and event rate of IMV and ICU in the study population.

From both meta‐analyses and meta‐regression, we excluded studies with preselected population, specifically SARS‐CoV‐2‐infected patients with certain coexisting diseases or conditions that potentially cause malnutrition (e.g., cystic fibrosis) or significantly influence body weight (pregnancy, paediatric population).

### 2.3 Data extraction

Two independent review authors extracted data from the eligible studies into a standardized data collection form. All disagreements were resolved by an independent third author. The following data were extracted from each included study: authors, publication year, digital object identifier, study site, study design, protocol number, age, gender distribution, number of patients in each reported BMI range, number of patients with IMV in each reported BMI range, number of patients with ICU admission in each reported BMI range, odds ratios for IMV and ICU admission regarding BMI groups.

### 2.4 BMI categories

We defined BMI classes based on the World Health Organization (WHO) guidelines.^17^ We included Asian and Caucasian populations in the same analysis—with their corresponding BMI cut off—only in meta‐analyses comparing patients with obesity and patients without obesity. We applied different cut‐off values for obesity in Asian‐Pacific (obesity >25 kg/m^2^) and Caucasian (obesity >30 kg/m^2^) population.^17^

### 2.5 Risk of bias assessment

Based on the recommendations of Cochrane Prognosis Methods Group (PMG), a modified version of the Quality in Prognostic Studies (QUIPS) tool was used by the two independent review authors to assess the quality of the studies included.^18^ Any disagreement was resolved by a third party.

### 2.6 Statistical analysis

We performed all meta‐analytic calculations with Comprehensive Meta‐Analysis (Version 3) statistical software (Biostat, Inc., Engelwood, MJ, USA). For data synthesis, we used the methods recommended by the working group of the Cochrane Collaboration. Pooled odds ratios (ORs) with their 95% confidence intervals (CIs) were calculated from raw data of the articles except for one article,^19^ where the given OR and the CI were used. The random effect model with the estimation of DerSimonian and Laird was used in all cases.^20^ Heterogeneity was tested using Cochrane's *Q* and the *I* ^2^ statistics. *I* ^2^ statistic represents the percentage of the total variability across studies: 30% to 60%, 50% to 90% and 75% to 100% corresponded to moderate, substantial and considerable degrees of heterogeneity, based on the Cochrane's handbook for Systematic Reviews of Interventions.^21^ We considered the Q test significant if *P* < 0.1. Publication bias was examined by visual inspection of funnel plots.

To examine the effect of BMI on the event rate of IMV, meta‐regression was applied. We examined if the regression coefficient is equal to zero. We assessed R2 analogue, which is the explained variances of the models. We calculated corresponding *P* values and *I* ^2^‐test values.

## 3 RESULTS

### 3.1 Results of search and selection

**FIGURE 1:**
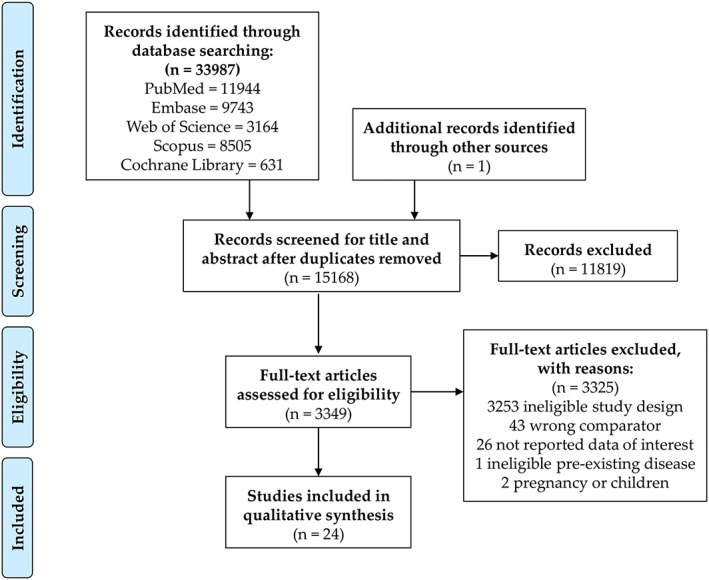
Preferred Reporting in Systematic Reviews and Meta‐analyses (PRISMA) flowchart showing the selection process

We described the selection process in detail in the PRISMA flow chart (Figure 1). We identified a total of 33,987 records, 24 of which were included in the qualitative and quantitative syntheses. Nine studies were included in the meta‐analyses, and 20 articles in the meta‐regression. Five studies contained data for both the meta‐analysis and the meta‐regression.

### 3.2 Characteristics of studies included

#### 3.2.1 Meta‐analysis

The characteristics of the studies included in the meta‐analyses are presented in Table 1 and Table S2. Six studies with 2,770 patients reported on ICU admission, the proportion of which ranged from 9% to 43%. Five studies with 509 patients reported on IMV requirement, the proportion of which ranged from 58% to 78%.

**TABLE 1:** Characteristics of the included studies

Study (country)	Cohort type	N^0^ of patients (female %)	Age^b^	BMI categories (N^0^)			Event	Event N^0^ (%)
				Normal (<25)	Overweight (25‐30)	Obese (>30)		
Nonobese vs. obese^a^								
Hu L et al (China)	Retrospective	294 (49%)	61	229	65		ICU admission	25 (9%)
Itelman E et al (Israel)	Retrospective	162 (35%)	52	131	31		ICU admission	26 (16%)
Kalligeros M et al (USA)	Retrospective	103 (38%)	60	44	59		ICU admission	44 (43%)
Lighter J et al (USA)	Retrospective	1759 (NR)	NR	1,010	749		ICU admission	431 (25%)
Lodigiani C et al (Italy)	Retrospective	361 (32%)	66	274	87		ICU admission	57 (16%)
Ong S et al (Singapore)	Retrospective	91 (NR) 27 (NR)	55	51 12	40 15		ICU admission IMV need	27 (30%) 16 (59%)
Normal weight vs. overweight or obese								
Bhatraju PK et al (USA)	Retrospective	23 (38%)	64	3	7	13	IMV need	18 (78%)
Caussy C et al (France)	Retrospective	291 (NR)	NR	74	121	96	IMV need	170 (58%)
Kalligeros M et al (USA)	Retrospective	44 (34%)	60	5	14	25	IMV need	29 (66%)
Simonnet A et al (France)	Retrospective	124 (27%)	60	17	48	59	IMV need	89 (72%)

*Note.* All studies were conducted in 2020.

Abbreviations: ICU, intensive care unit; IMV, invasive mechanical ventilation; NR = not reported.

^a^ Different cut‐off values were used to define obesity in Asian and Caucasian population.

^b^ Mean or median.

#### 3.2.2 Meta‐regression

The characteristics of the studies included in the meta‐regression analysis are presented in Table S3.

### 3.3 Association between BMI and ICU admission

#### 3.3.1 Qualitative and quantitative synthesis comparing BMI classes

Lodigiani et al reported 15.4% versus 15.9% ICU admission ratio among patients with BMI less than 25 and with BMI greater than or equal to 25, respectively.^22^ In another study, a higher ICU admission ratio was observed among patients with BMI values greater than or equal to 25 compared with patients with a BMI of 25 or lower (46.4% vs. 26.3%, respectively).^19^

**FIGURE 2:**
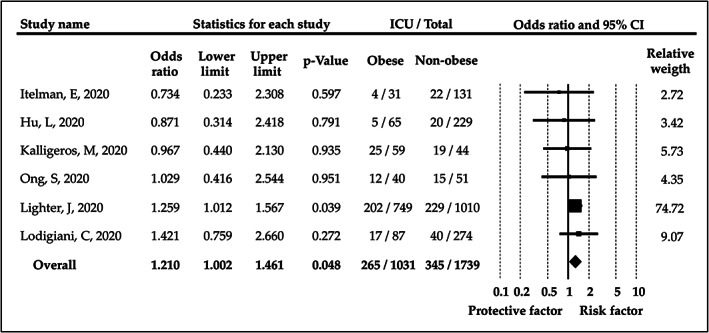
Odds ratios for intensive care unit admission in patients with obesity versus patients without obesity. BMI, body mass index; CI, confidence interval; ICU, intensive care unit

Our pooled analysis of six studies showed that COVID‐19 patients with obesity have a significantly higher risk for ICU admission (OR = 1.21, CI: 1.002‐1.46; *I* ^2^ = 0.0%).^19^, ^22^, ^23^, ^24^, ^25^, ^26^ This result is depicted in Figure 2.

There were not enough studies to compare ICU admission ratios between different BMI ranges.

#### 3.3.2 Multivariate analyses

Kalligeros et al evaluated the association between different BMI ranges and ICU admission with multivariate logistic regression analysis. They found that BMI greater than or equal to 35 carries a six times higher risk for ICU admission compared with BMI lower than 25 (OR = 6.16, CI: 1.42‐26.66).^19^ Their results are summarized in Table S4.

#### 3.3.3 Meta‐regression

Because of insufficient data, we could not perform meta‐regression to assess the correlation between BMI and ICU admission. The BMI values of the eligible studies and the corresponding ICU admission ratios are presented in Table S2.

### 3.4 Association between BMI and IMV requirement

#### 3.4.1 Qualitative and quantitative synthesis comparing BMI classes

We found that IMV is significantly more likely to occur in patients with BMI greater than or equal to 25 compared with those with BMI lower than 25 (OR = 2.63, CI: 1.64‐4.22; *I* ^2^ = 0.0%).^15^, ^27^, ^28^ The result of this analysis is shown in Figure S1.

**FIGURE 3:**
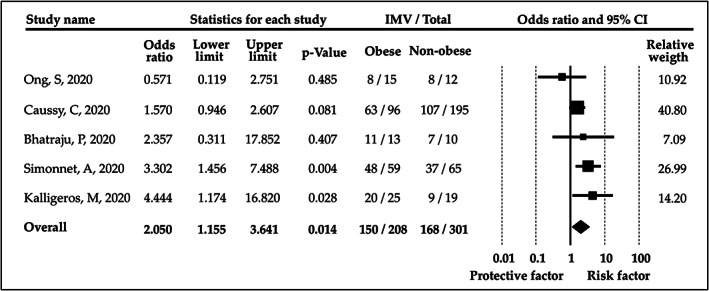
Odds ratios for invasive mechanical ventilation (IMV) in patients with obesity versus patients without obesity. CI, confidence interval

The comparison of patients with obesity and without obesity revealed an increased risk for IMV among patients with obesity (OR = 2.05, CI: 1.16‐3.64; *I* ^2^ = 34.86%).^15^, ^19^, ^25^, ^27^, ^28^ The forest plot displaying this result is found in Figure 3.

**FIGURE 4:**
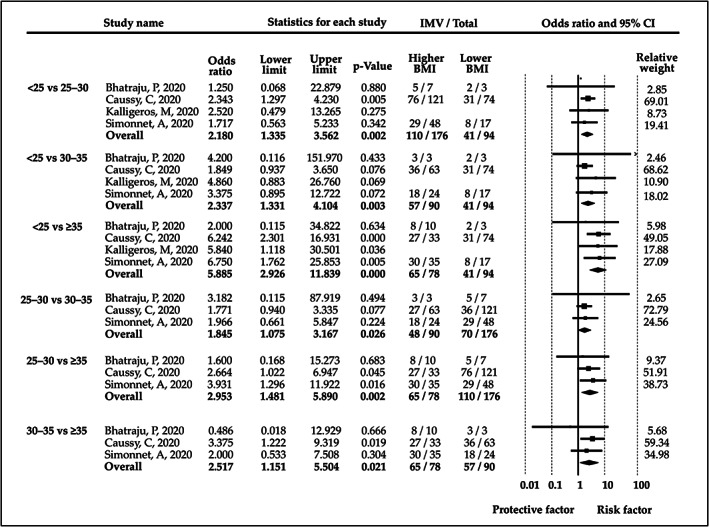
Odds ratios for invasive mechanical ventilation (IMV) between patient groups with different BMI ranges (<25, 25‐30, 30‐35 and ≥35). BMI = body mass index, CI = confidence interval

There were enough studies to statistically compare the reported BMI ranges (<25, 25‐30, 30‐35 and ≥35) with each other. We found that the higher BMI ranges always carry a significantly higher risk for IMV compared with the categories with a lower range. The BMI subgroup analyses are shown in Figure 4.

#### 3.4.2 Multivariate analyses

Kalligeros et al assessed the association between different BMI ranges and IMV with multivariate logistic regression analysis. They found that BMI greater than or equal to 35 carries a six times higher risk for IMV compared with BMI lower than 25.^19^ Simonnet et al reported an increased risk for IMV among patients with higher BMI using multivariate logistic regression analysis.^15^ They found that BMI ranges of 25 to 30, 30 to 35 and BMI greater than or equal to 35 carry a significantly higher risk for IMV compared to patients with BMI lower than 25. The results of these studies are summarized in Table S4.

#### 3.4.3 Meta‐regression

Meta‐regression scatter plot representing the correlation between BMI and IMV is shown in Figure S2.^15^, ^28^, ^29^, ^30^, ^31^, ^32^, ^33^, ^34^ No correlation was found (*P* = 0.8890, *Q* = 0.02, *I* ^2^ = 63.39%, *p*_heterogeneity_ = 0.0078). However, the analysis provides information only for a narrow BMI range.

### 3.5 Risk of bias assessment and publication bias

Funnel plots indicating the possibility of publication bias are found in Figures S3 and S4. Egger's tests could not be performed to detect publication bias due to the number of included studies in each hypothesis. Visual assessment of the funnel plots did not imply asymmetry.

The results of the risk of bias assessment of individual studies are shown in Table S5.

### 3.6 Assessment of heterogeneity

There was no statistical heterogeneity in the meta‐analyses except for comparison of patients with obesity and without obesity regarding IMV requirement (*I* ^2^ = 34.86%, moderate level of statistical heterogeneity). The *P* values related to *I* ^2^ were over 0.01.

## 4 DISCUSSION

According to our meta‐analysis and systematic review, patients with higher BMI have a greater risk for ICU admission and especially for IMV in all comparisons.

The knowledge about the role of obesity in the disease course of respiratory tract infections is limited. Previous studies suggested that obesity might be associated with poor prognosis in COVID‐19.^35^, ^36^, ^37^ Subsequent data have called attention to the higher need for intensive care and mechanical ventilation.^15^, ^19^, ^28^ However, there is no previous meta‐analysis in this topic, and the available studies cannot lead to definitive conclusions because of the low sample sizes and their retrospective nature. Nevertheless, gathering knowledge of the clinical characteristics of these patients is of utmost importance as this could facilitate adequate management and resource allocation.^38^

### 4.1 Obesity and ICU admission

Due to scarce data, we could not perform a conclusive meta‐regression to assess the correlation between BMI and ICU admission, although it is well known that obesity carries enhanced risk of chronic diseases that could contribute to the increased need for ICU admission and unfavourable outcomes in COVID‐19 patients. Unfortunately, initial studies neglected the anthropometric data of patients.^39^

Nevertheless, we found in our pooled analysis that obesity is associated with a higher risk for ICU admission in COVID‐19 patients. Since the lower endpoint of the CI in this analysis is close to the zero effect, we feel that it needs confirmation by further studies. This would be particularly important because limited ICU capacity has caused great concern worldwide. Indeed, leading officials of intensive care societies worldwide have proposed to increase ICU capacity because health care should provide intensive care to everyone who needs it.^40^

The association between obesity and poor clinical outcomes is certainly multifactorial since obesity itself is widely associated with several prognostic factors.^7^ In COVID‐19 patients, hypertension, diabetes and cardiovascular problems are the most common comorbidities^19^ that may share similar pathways with obesity related to the renin‐angiotensin system (RAS).^41^ Obesity modulates the RAS activity,^42^ which can lead to pathological processes in COVID‐19.^43^ In this connection, another interesting point was proposed by Lighter et al, namely that a BMI of 35 and above is a risk factor for ICU admission in patients aged less than 60 years, but not in the elderly.^26^ Ong et al presented similar findings on IMV requirement.^25^ According to the previous studies, obesity seems to increase general mortality risk at older ages, but to a lesser extent than at younger ages.^44^, ^45^

### 4.2 Obesity and the need for IMV

Among COVID‐19 patients admitted to the ICU, 40% to 100% of patients require IMV.^33^, ^46^, ^47^ Excessive body mass and obstructive sleep apnea might complicate different forms of respiratory support and endotracheal intubation.^48^, ^49^ Prolonged endotracheal intubation time, which is more frequently present in patients with obesity, also increases the risk of infection of the medical staff.^50^ These are of particular importance considering that we have shown a greater risk for IMV in patients with higher BMI in all comparisons.

In general, several studies in ICU have shown that patients with higher BMI require IMV more frequently and for a longer period.^51^, ^52^ This could be partly explained by the reduced pulmonary reserves, anatomical alterations of the chest wall; hence, it is not surprising that these patients have a higher risk for progressing into ARDS.^53^, ^54^

Moreover, obesity may also alter immune response. Protective immune functions might be disabled; for instance, vaccination success can be poor in the case of obesity.^55^ Both hyperinflammation and enhanced coagulation have been reported as frequent finding in both COVID‐19 and obesity.^56^, ^57^ These features and findings in COVID‐19 patients may explain our results, but until specifically investigated, this remains a hypothesis.

### 4.3 Strengths

To our knowledge, this is the first meta‐analysis about this topic despite its importance. We followed a rigorous methodology, and besides meta‐analyses, we also performed meta‐regression and the assessment of publication bias. No considerable heterogeneity was detected in any analyses. To assess the risk‐increasing effect of obesity as accurately as possible, we also conducted analyses that compare BMI ranges with each other.

### 4.4 Limitations

A crucial limitation is that our results are from a sparse number of retrospective studies. In addition, our analysis can be limited by the different strategies of different hospitals regarding ICU admission and an indication of IMV, which has been scarcely defined across the included studies. Besides, the BMI distribution can also be largely different between Asian and Caucasian populations. We sought to correct this in our analysis, but the limitation could not be completely avoided.

### 4.5 Implications for future practice

Based on our findings, it is highly recommended to measure anthropometric parameters of patients and include BMI in the development of a specific COVID‐19 risk assessment score in the future. As patients with higher BMI have a greater risk for ICU admission and IMV, patients with obesity may need special monitoring and earlier escalation of treatment. Therefore, it is important to amend guidelines for COVID‐19 risk stratification and treatment with special attention to patients with obesity. Besides, prevention might also be important. Returning waves of COVID‐19 pandemic cannot be excluded; therefore, it remains important to support weight loss and regular physical activity and also to elaborate vaccination programmes with special regard to the higher risk groups including individuals with elevated BMI.

### 4.6 Implications for future research

Because of the limited information on this topic, it should be subject to future studies. Multivariate analyses should be conducted to examine whether obesity is an independent risk factor, as it has been suggested in the case of H1N1 influenza.^13^ Anthropometric parameters of patients with COVID‐19 should be included in future studies.

Conducting further basic research would be crucial for a better understanding of the pathogenesis of COVID‐19 in patients with obesity. Literature suggests that adipose tissue may serve as a reservoir for certain pathogens (e.g., Influenza A virus and *Mycobacterium tuberculosis* ).^58^ It would be worth examining this in terms of COVID‐19 as well.

## 5 CONCLUSION

In summary, our meta‐analysis and systematic review revealed that obesity is a significant risk factor for ICU admission and particularly for IMV requirement in COVID‐19. It may serve as a clinical predictor for risk stratification models; therefore, measurement of anthropometric and metabolic parameters in COVID‐19 would be crucial. Patients with obesity should be closely monitored and might need escalation of therapy earlier to avoid unfavourable clinical outcomes. As returning waves of the pandemic are expected, improvement of guidelines for patients with obesity is highly recommended.

## CONFLICTS OF INTEREST

The authors have no conflict of interest to declare.

## FUNDING INFORMATION

This study was funded by the Economic Development and Innovation Operational Programme (European Regional Development Fund) within the framework of Programme Széchenyi 2020 (GINOP‐2.3.2‐15‐2016‐00048 – STAY ALIVE) and the Human Resources Development Operational Programme (European Regional Development Fund, EFOP 3.6.2‐16‐2017‐00006 – LIVE LONGER) and the Medical School of University of Pécs.

## Supporting information


**Figure S1.** Forest plot comparing patients with overweight or obesity to patients with normal weight regarding invasive mechanical ventilation. IMV=invasive mechanical ventilation, CI=confidence interval
**Figure S2.** Meta‐regression assessing the correlation between the body mass index and invasive mechanical ventilation, BMI=body mass index
**Figure S3.** Funnel plot assessing the publication bias of the meta‐analysis that compares non‐obese and obese patients regarding intensive care unit.
**Figure S4.** Funnel plot assessing the publication bias of the meta‐analysis that compares non‐obese and obese patients regarding invasive mechanical ventilation.
**Table S1.** Supporting Information
**Table S2.** Eligibility criteria in each included study in the meta‐analysis
**Table S3.** Characteristics of the studies included in the meta‐regression
**Table S4.** Study‐level data on multivariate analysis
**Table S5.** Risk of bias assessment with QUIPS tool
