# Supplementary material for: Obesity is a risk factor for developing critical condition in COVID‐19 patients: A systematic review and meta‐analysis
Source: Obes Rev. 2020 Jul 19;21(10):e13095. doi: 10.1111/obr.13095 (PMC7404429; doi:10.1111/obr.13095)
Supplement: Supplementary file 1 — Figure S1. Forest plot comparing patients with overweight or obesity to patients with normal weight regarding invasive mechanical ventilation. IMV=invasive mechanical ventilation, CI=confidence interval Figure S2. Meta‐regression assessing the correlation between the body mass index and invasive mechanical ventilation, BMI=body mass index Figure S3. Funnel plot assessing the publication bias of the meta‐analysis that compares non‐obese and obese patients regarding intensive care unit. Figure S4. Funnel plot assessing the publication bias of the meta‐analysis that compares non‐obese and obese patients regarding invasive mechanical ventilation. Table S1. Supporting Information Table S2. Eligibility criteria in each included study in the meta‐analysis Table S3. Characteristics of the studies included in the meta‐regression Table S4. Study‐level data on multivariate analysis Table S5. Risk of bias assessment with QUIPS tool [file OBR-21-0-s001.pdf]

Obesity is a Risk Factor for Developing Critical Condition in COVID-19 Patients: A Systematic Review and Meta-analysis

Mária Földi[1,2,3] foldimarcsi4@gmail.com, Nelli Farkas[1,2] farkas.nelli@gmail.com, Szabolcs Kiss[1,2,3] kissszabolcs1995@gmail.com, Noémi Zádori[1,2] znoeemi@gmail.com, Szilárd Váncsa[1,2] vancsaszilard@gmail.com, Lajos Szakó[1,2] szaklaj@gmail.com, Fanni Dembrovszky[1,2] dembrovszky.f@gmail.com, Margit Solymár[1,2] margit.solymar@aok.pte.hu, Eszter Bartalis[1,4] 19bartaliseszter95@gmail.com, Zsolt Szakács[1,2] szaki92@gmail.com, Petra Hartmann[5] drhartmann.petra@gmail.com, Gabriella Pár[6] par.gabriella@pte.hu, Bálint Erőss[1,2] eross.balint@pte.hu, Zsolt Molnár[1,2,7] zsoltmolna@gmail.com, Péter Hegyi[1,2] hegyi2009@gmail.com, and Andrea Szentesi[1,2,8*] szentesiai@gmail.com

[1]Institute for Translational Medicine, Medical School, University of Pécs, Pécs, Hungary;
[2]Szentágothai Research Centre, University of Pécs, Pécs, Hungary
[3]Doctoral School of Clinical Medicine, University of Szeged, Szeged, Hungary
[4]University of Medicine, Pharmacy, Science and Technology of Targu Mures, Targu Mures, Romania; (EB)
[5]Institute of Surgical Research, University of Szeged, Szeged, Hungary;
[6]Division of Gastroenterology, First Department of Medicine, Medical School, University of Pécs, Pécs, Hungary
[7]Department of Anesthesiology and Intensive Therapy, Poznan University for Medical Sciences, Poznan, Poland
[8]First Department of Medicine, University of Szeged, Szeged, Hungary

*****Correspondence: Andrea Szentesi, Operative Director
Adress: Center for Translational Medicine, University of Pécs 12 Szigeti street, H-7624 Pécs, Hungary, Tel.: +36 30 342 1481, E-mail: szentesiai@gmail.com; Phone: +36-30-342-1481

Study name	Statistics for each study				IMV / Total		Relative weight
	Odds ratio	Lower limit	Upper limit	p-Value	Overweight and obesity	Normal weight	
Bhatraju, P, 2020	2.000	0.143	27.990	0.607	16 / 20	2 / 3	3.19
Caussy, C, 2020	2.472	1.442	4.236	0.001	139 / 217	31 / 74	76.63
Simonnet, A, 2020	3.505	1.227	10.014	0.019	81 / 107	8 / 17	20.17
**Overall**	**2.634**	**1.644**	**4.221**	**0.000**	**236 / 344**	**41 / 94**	

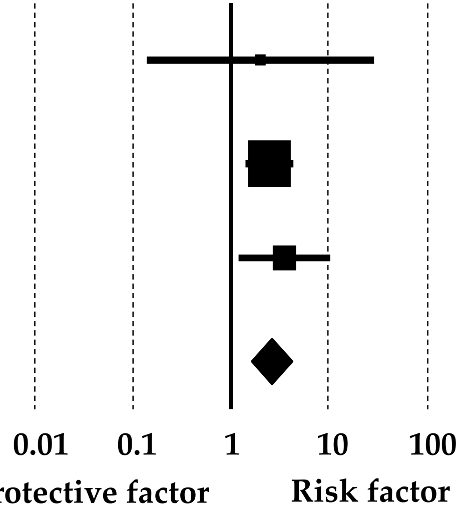

Figure S1: Forest plot comparing patients with overweight or obesity to patients with normal weight regarding invasive mechanical ventilation. IMV=invasive mechanical ventilation, CI=confidence interval

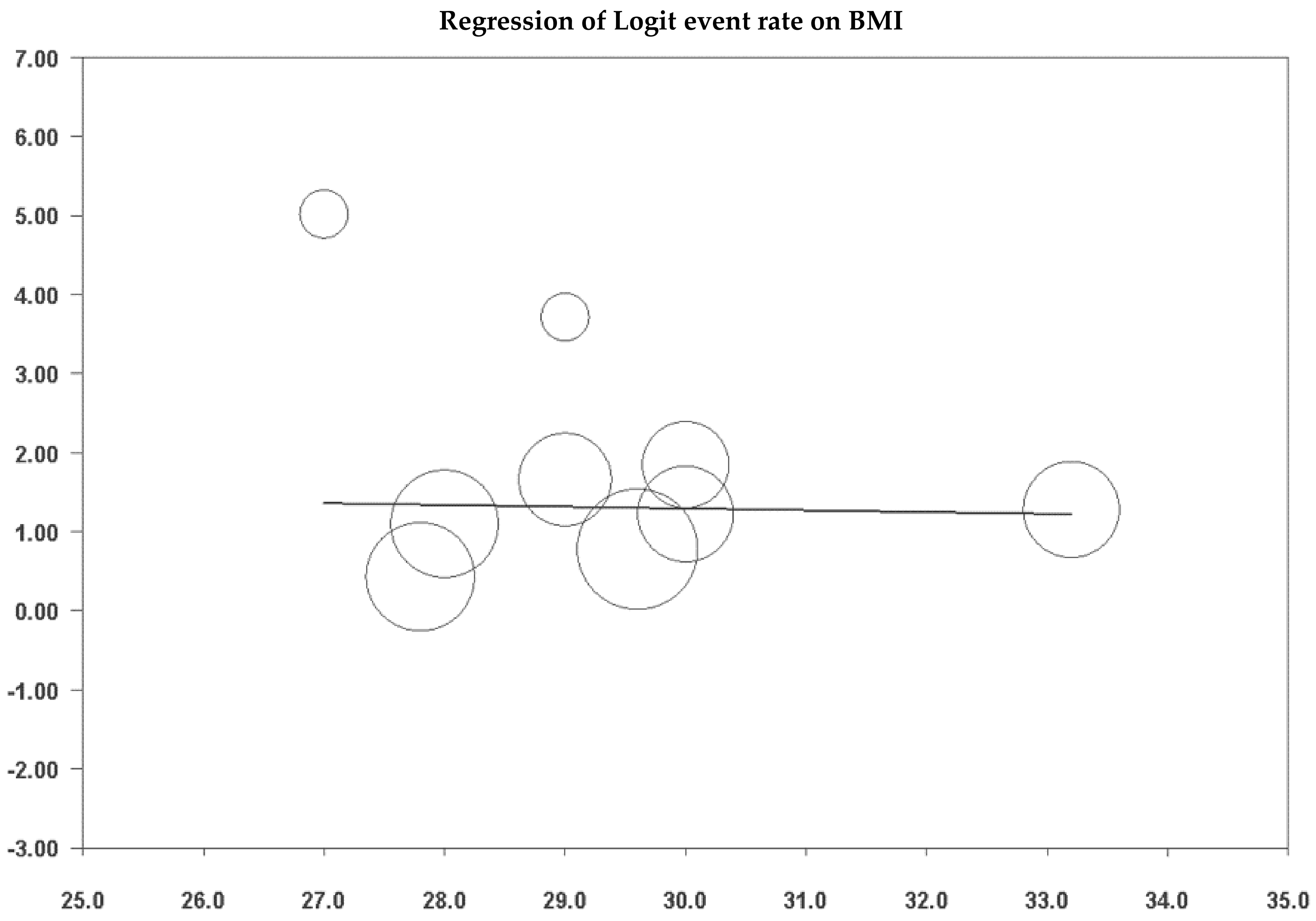

Figure S2: Meta-regression assessing the correlation between the body mass index and invasive mechanical ventilation, BMI=body mass index

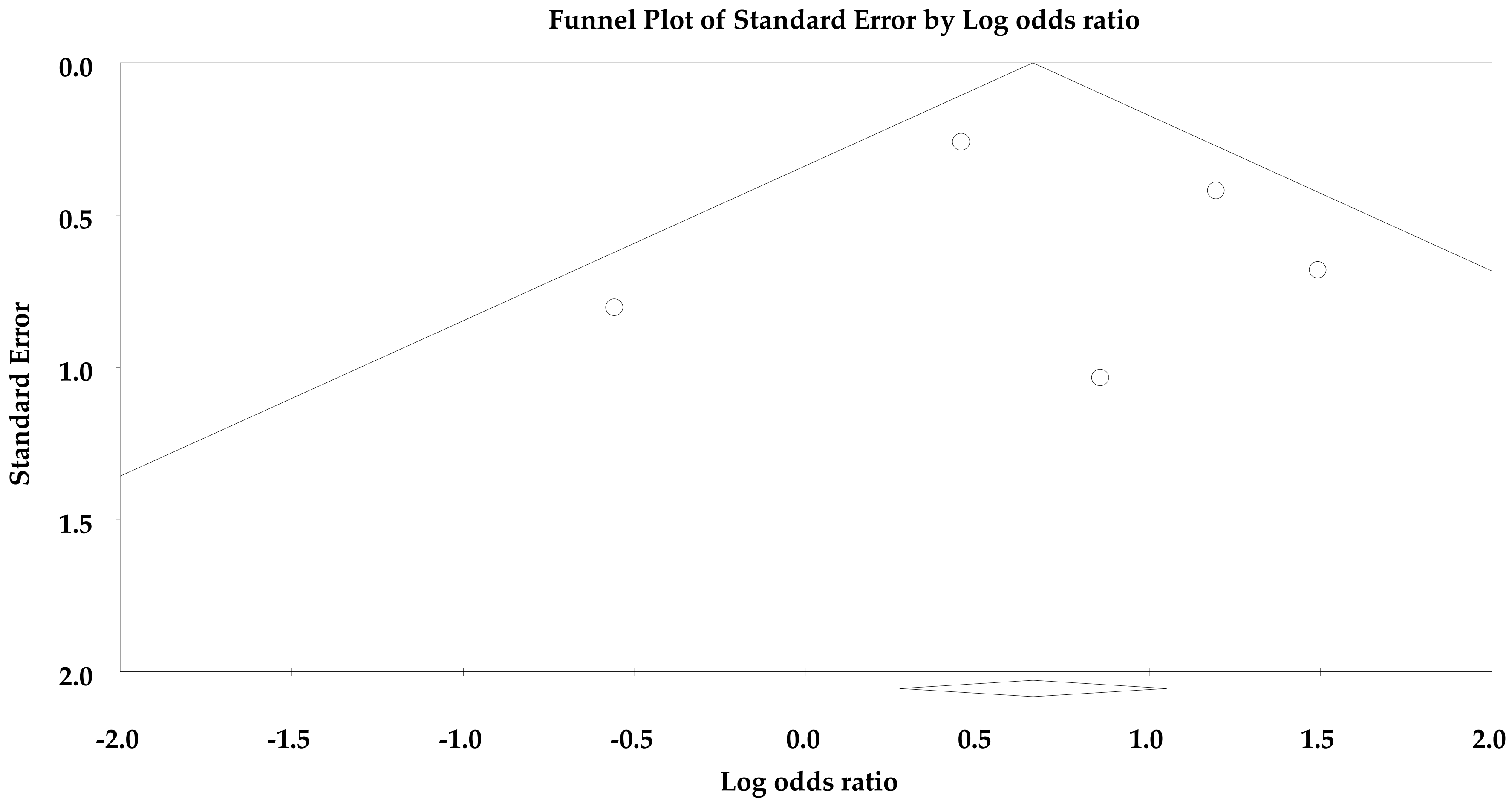

Figure S3: Funnel plot assessing the publication bias of the meta-analysis that compares non-obese and obese patients regarding intensive care unit.

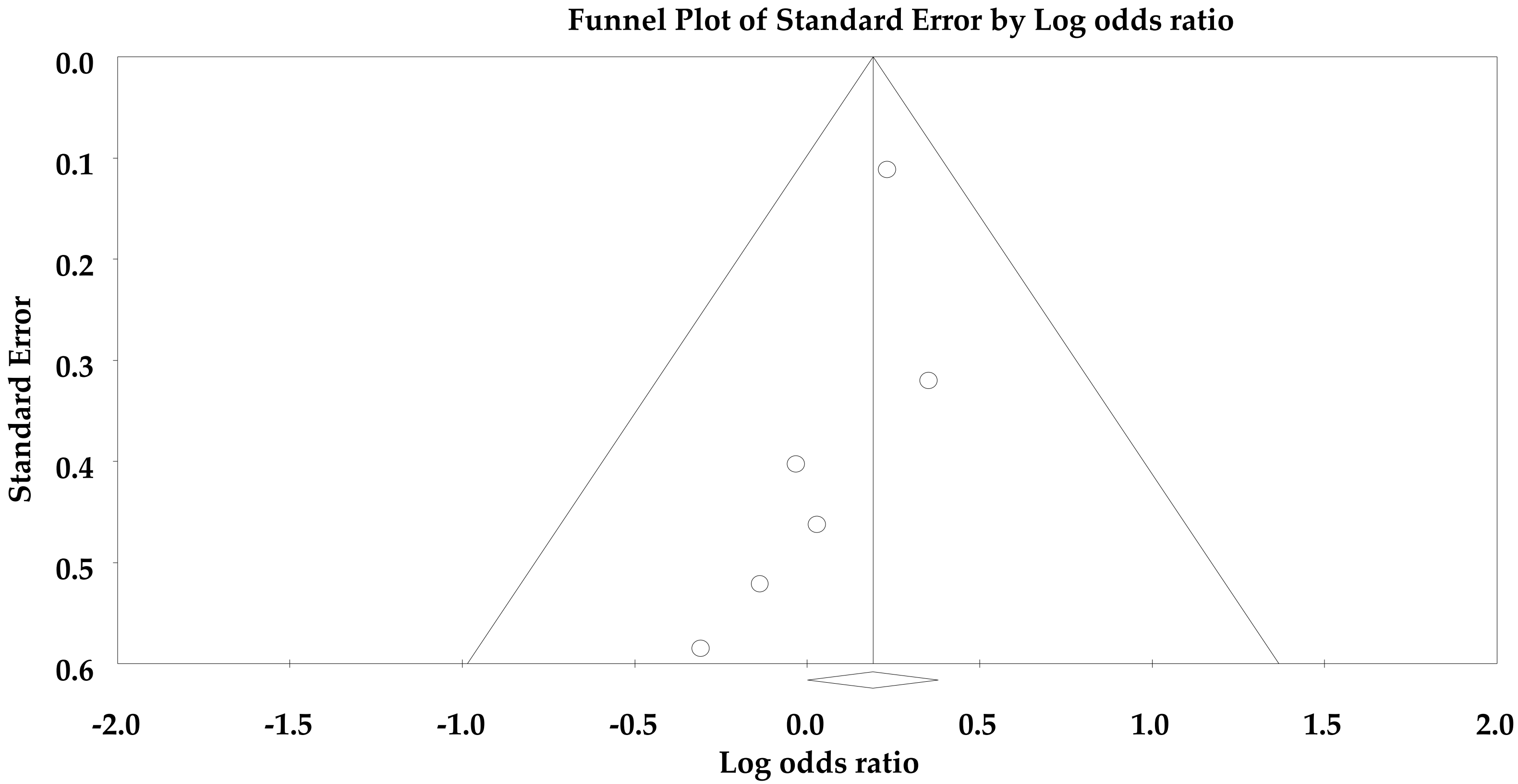

Figure S4: Funnel plot assessing the publication bias of the meta-analysis that compares non-obese and obese regarding invasive mechanical ventilation.

Table S1

PRISMA 2009 Checklist

Section/topic	#	Checklist item	Reported on page #
**TITLE**			
Title	1	Identify the report as a systematic review, meta-analysis, or both.	1/14
**ABSTRACT**			
Structured summary	2	Provide a structured summary including, as applicable: background; objectives; data sources; study eligibility criteria, participants, and interventions; study appraisal and synthesis methods; results; limitations; conclusions and implications of key findings; systematic review registration number.	1/14
**INTRODUCTION**			
Rationale	3	Describe the rationale for the review in the context of what is already known.	2/14
Objectives	4	Provide an explicit statement of questions being addressed with reference to participants, interventions, comparisons, outcomes, and study design (PICOS).	2/14
**METHODS**			
Protocol and registration	5	Indicate if a review protocol exists, if and where it can be accessed (e.g., Web address), and, if available, provide registration information including registration number.	2/14
Eligibility criteria	6	Specify study characteristics (e.g., PICOS, length of follow-up) and report characteristics (e.g., years considered, language, publication status) used as criteria for eligibility, giving rationale.	2–3/14
Information sources	7	Describe all information sources (e.g., databases with dates of coverage, contact with study authors to identify additional studies) in the search and date last searched.	2/14
Search	8	Present full electronic search strategy for at least one database, including any limits used, such that it could be repeated.	2/14
Study selection	9	State the process for selecting studies (i.e., screening, eligibility, included in systematic review, and, if applicable, included in the meta-analysis).	2–3/14
Data collection process	10	Describe method of data extraction from reports (e.g., piloted forms, independently, in duplicate) and any processes for obtaining and confirming data from investigators.	3/14
Data items	11	List and define all variables for which data were sought (e.g., PICOS, funding sources) and any assumptions and simplifications made.	3/14
Risk of bias in individual studies	12	Describe methods used for assessing risk of bias of individual studies (including specification of whether this was done at the study or outcome level), and how this information is to be used in any data synthesis.	3/14
Summary measures	13	State the principal summary measures (e.g., risk ratio, difference in means).	2/14
Synthesis of results	14	Describe the methods of handling data and combining results of studies, if done, including measures of consistency (e.g., I^2) for each meta-analysis.	3/14

Table S1

PRISMA 2009 Checklist

Section/topic	#	Checklist item	Reported on page #
Risk of bias across studies	15	Specify any assessment of risk of bias that may affect the cumulative evidence (e.g., publication bias, selective reporting within studies).	NA
Additional analyses	16	Describe methods of additional analyses (e.g., sensitivity or subgroup analyses, meta-regression), if done, indicating which were pre-specified.	3/14
**RESULTS**			
Study selection	17	Give numbers of studies screened, assessed for eligibility, and included in the review, with reasons for exclusions at each stage, ideally with a flow diagram.	4/14
Study characteristics	18	For each study, present characteristics for which data were extracted (e.g., study size, PICOS, follow-up period) and provide the citations.	4–5/14
Risk of bias within studies	19	Present data on risk of bias of each study and, if available, any outcome level assessment (see item 12).	(8/14) + Table S5
Results of individual studies	20	For all outcomes considered (benefits or harms), present, for each study: (a) simple summary data for each intervention group (b) effect estimates and confidence intervals, ideally with a forest plot.	6–8/14 + Figure S1, Table S3–4
Synthesis of results	21	Present results of each meta-analysis done, including confidence intervals and measures of consistency.	6–8/14
Risk of bias across studies	22	Present results of any assessment of risk of bias across studies (see Item 15).	NA
Additional analysis	23	Give results of additional analyses, if done (e.g., sensitivity or subgroup analyses, meta-regression [see Item 16]).	6-8/14
**DISCUSSION**			
Summary of evidence	24	Summarize the main findings including the strength of evidence for each main outcome; consider their relevance to key groups (e.g., healthcare providers, users, and policy makers).	8–9/14
Limitations	25	Discuss limitations at study and outcome level (e.g., risk of bias), and at review-level (e.g., incomplete retrieval of identified research, reporting bias).	9/14
Conclusions	26	Provide a general interpretation of the results in the context of other evidence, and implications for future research.	10/14
**FUNDING**			
Funding	27	Describe sources of funding for the systematic review and other support (e.g., supply of data); role of funders for the systematic review.	10/14

From: Moher D, Liberati A, Tetzlaff J, Altman DG, The PRISMA Group (2009). Preferred Reporting Items for Systematic Reviews and Meta-Analyses: The PRISMA Statement. PLoS Med 6(7): e1000097. doi:10.1371/journal.pmed1000097

Table S2. Eligibility criteria in each included study in the meta-analysis

Study	Outcome	Definition	Follow-up	Eligibility
Bhatraju PK et al	IMV need	Acute respiratory distress syndrome (ARDS) was defined as acute-onset hypoxemia (the ratio of the partial pressure of arterial oxygen to the fraction of inspired oxygen [Pao2:Fio2], <300) with bilateral pulmonary opacities on chest imaging that were not fully explained by congestive heart failure or other forms of volume overload	23/03/2020	A confirmed case of Covid-19 was defined by a positive result on a reverse-transcriptase–polymerase-chain-reaction (RT-PCR) assay of a specimen collected on a nasopharyngeal swab. Only laboratory-confirmed cases were included. Pregnant women, prisoners, and children (those younger than 18 years of age) were excluded from the study
Caussy C et al	IMV need	Although we agree that invasive mechanical ventilation (IMV) can be considered as a reliable outcome for the severity of SARS-Cov-2, there is currently no guideline for the indication of IMV in the context of SRAS-Cov-2.	ND	NR
Hu L et al	ICU admission	Based on the clinical presentation at the time of admission, patients were categorized into one of three groups: non-severe, severe and critical.	10/03/2020	Diagnosis complied with the WHO interim guidance and the guidelines of COVID-19 diagnosis and treatment trial (5th edition), by the National Health Commission of the People's Republic of China.
Itelman E et al	ICU admission	We chose a simplified version to classify symptoms: mild disease included flu-like without clinical and imaging signs of pneumonia; moderate included pneumonia and hypoxemia; and severe included requiring intensive help for proper oxygenation (either high-flow oxygen delivery device or artificial ventilation, either non-invasive or invasive).	NR	NR
Kalligeros M et al	ICU admission;	ICU admission within the first 10 days of hospital admission with COVID-19. Our	NR	consecutive adult (≥ 18 years old) patients, who had a laboratory confirmed (using a reverse

	IMV need	secondary objective was to assess if the aforementioned factors are associated with the need for IMV during the first 10 days of hospital admission with COVID-19.		transcriptase–polymerase chain reaction assay) SARS-Cov-2 infection
Lighter J et al	ICU admission	Critical care was defined based on intensive care accommodation status or invasive ventilator documentation in our electronic health record.	NR	Patients who were PCR-positive for Covid-19
Lodigiani C et al	ICU admission	Patients requiring intensive care	NR	laboratory-proven COVID-19
Ong S et al	ICU admission IMV need	adverse outcomes analyzed were hypoxia requiring supplemental oxygen, ICU admission, mechanical ventilation, and mortality.	NR	laboratory confirmed COVID-19 (by polymerase chain reaction assay)
Peng YD et al	ICU admission	Critical (one of the following situations): respiratory failure requires mechanical ventilation; shock; combined with other organ failure requires intensive care unit (ICU)	NR	patients with combined cardiovascular diseases (hypertension, coronary heart disease and heart failure)
Simonnet A et al	IMV need	The primary outcome of this study was the prevalence of patients receiving invasive mechanical ventilation (IMV) following admission to intensive care. The use of IMV was determined when oxygen therapy (≥ 10 L/min) with target spO_2 (90-94%) was ineffective, and when respiratory rate was above 25/min, with signs of acute respiratory failure, despite maximal oxygen therapy.	06/04/2020	All patients were diagnosed with COVID-19 pneumonia according to World Health Organization interim guidance (11) with SARS symptoms characterized by dyspnea, increased respiratory frequency, decreased blood oxygen saturation, and need for oxygen support therapy for at least 6 L/min. Throat swab samples were obtained from all patients at admission and tested using real-time reverse transcriptase–polymerase chain reaction

Table S3. Characteristics of the studies included in the meta-regression

Study name	Study design	Country	Event	N0 of ALL patients	N0 of ALL patients with EVENT	BMI in the study						
						Mean	SD	Median	Range min	Range max	IQR min	IQR max
Alattar, R, 2020	RC	Qatar	IMV	25	21			29		27	34	
Bessiére, F, 2020	RC	France	IMV	40	30			28		25	33	
Bhatraju, P, 2020	RC	USA	IMV	23	18	33.2	7.2					
Cardoso, FS, 2020	RC	Portugal	IMV	20	20			29			26	32
Middeldrop, S, 2020	RC	Netherlands	IMV	75	75			27			24	31
Piva, S, 2020	RC	Italy	IMV	33	20			27.8			27	32.1
Poissy, J, 2020	RC	France	IMV	22	17			30	22	53		
Simonnet, 2020	RC	France	IMV	124	85			29.6			26.4	36.4
Spiezia, 2020	RC	Italy	IMV	22	19	30	6					
Cai, Q, Huang, D, 2020	RC	China	ICU	298	34			23.05			20.9	25.4
Fang, Z, 2020	RC	China	ICU	32	8			24.5			22.6	26.5
Huang, R, Zhu, R	RC	China	ICU	202	11			24.4			22.3	26.4
Ji, D, Qin, E	RC	China	ICU	202	1	24	2.8					
Li, X, 2020	RC	China	ICU	548	46			24.7			22.4	26.7
Peng, Y, 2020	RC	China	ICU	112	17			22			20	25
Wu, J, 2020	RC	China	ICU	280	83	24.1	3					
Haberman, R, 2020	RC	USA	ICU	14	1	30.8	8					
Itelman, E, 2020	RC	Israel	ICU	162	26			27.3			23.9	31.2
Mercuno, NJ, 2020	RC	Israel	ICU	90	30	31.5	6.6					
Middeldrop, S, 2020	RC	Netherlands	ICU	198	75			27			24	31

Abbreviations: RC=retrospective, BMI=body mass index, IMV=invasive mechanical ventilation, ICU=intensive care unit, SD=standard deviation, IQR=interquartile range

Table S4. Study-level data on multivariate analysis

Study name (country)	Investigated event	Risk factor (reference: BMI<25)	Adjusted factors in multivariate logistic regression	OR (95% CI)	p value
Kalligeros et al. (USA) (*103 patients in the study*)	ICU admission	BMI 25-29.9	age, race, gender	2.14 (0.58-7.88)	0.25
Kalligeros et al. (USA)	ICU admission	BMI 25-29.9	age, race, gender, diabetes, hypertension, lung disease[1], heart disease[2]	2.27 (0.59-8.83)	0.235
Kalligeros et al. (USA)	ICU admission	BMI 30-34.9	age, race, gender	2.56 (0.64-10.1)	0.1
Kalligeros et al. (USA)	ICU admission	BMI 30-34.9	age, race, gender, diabetes, hypertension, lung disease[1], heart disease[2]	2.65 (0.64-10.95)	0.178
Kalligeros et al. (USA)	ICU admission	BMI≥35	age, race, gender	**6.16 (1.42-26.66)**	**0.015**
Kalligeros et al. (USA)	ICU admission	BMI≥35	age, race, gender, diabetes, hypertension, lung disease[1], heart disease[2]	**5.39 (1.13-25.64)**	**0.034**
Kalligeros et al. (USA) (*34 patients in the study*)	IMV	BMI 25-29.9	age, race, gender	2.64 (0.48-14.4)	0.262
Kalligeros et al. (USA)	IMV	BMI 25-29.9	age, race, gender, diabetes, hypertension, lung disease[1], heart disease[2]	3.70 (0.60-22.87)	0.159
Kalligeros et al. (USA)	IMV	BMI 30-34.9	age, race, gender	5.28 (0.91-30.48)	0.063
Kalligeros et al. (USA)	IMV	BMI 30-34.9	age, race, gender, diabetes, hypertension, lung disease[1], heart disease[2]	**6.85 (1.05-44.82)**	**0.045**

Kalligeros et al. (USA)	IMV	BMI≥35	age, race, gender	**8.19 (1.36-49.13)**	**0.021**
Kalligeros et al. (USA)	IMV	BMI≥35	age, race, gender, diabetes, hypertension, lung disease[1], heart disease[2]	**9.99 (1.39-71.69)**	**0.022**
Simonnet et al. (France) (*124 patients in the study*)	IMV	BMI 25-30.0	age, diabetes, hypertension	1.69 (0.52-5.48)	0.22
Simonnet et al. (France)	IMV	BMI 30-35	age, diabetes, hypertension	3.45 (0.83-12.31)	0.48
Simonnet et al. (France)	IMV	BMI≥35	age, diabetes, hypertension	**7.36 (1.63-33.14)**	**0.021**

[1]: COPD, asthma, interstitial lung disease and pulmonary hypertension; [2]heart failure, coronary artery disease and cardiomyopathy
Abbreviations: IMV=invasive mechanical ventilation, ICU=intensive care unit, BMI=body mass index, CI=confidence interval

Table S5. Risk of bias assessment with QUIPS tool

Study	Study participation	Study attrition	Obesity	Outcome		Study confounding	Statistical analysis reporting
				Mechanical ventilation	Intensive care unit adm.		
Alattar R, Ibrahim T	+	N/A	+	?	?	?	+
Bessiere F, Roccia H	+	N/A	+	?	?	?	+
Bhataju PK, Ghassemieh BJ	+	N/A	+	?	?	?	+
Cai Q, Huang D	+	N/A	+	?	?	–	+
Cardoso FS, Pereira R	?	N/A	+	?	?	?	+
Caussy C, Wallet F	?	N/A	+	?	N/A	?	+
Fang Z, Zhang Y	+	N/A	+	?	?	?	+
Haberman R, Axelrad J	–	?	+	?	?	–	+
Hu L, Chen S	+	N/A	+	?	N/A	–	+
Huang R, Zhu Li	+	N/A	+	?	?	?	+
Itelman E, Waaserstrum Y	?	N/A	+	?	?	?	+
Ji D, Qin E	+	+	+	?	?	+	+
Kalligeros M, Shehadeh F	+	N/A	+	?	?	+	+
Li X, Xu S	+	+	+	?	N/A	?	+
Lighter J, Phillips M	+	N/A	+	?	+	?	+
Lodigiani C, Lapichino G	+	N/A	+	?	?	?	+
Mercuro NJ, Yen CF	+	N/A	+	?	?	–	+
Middeldorp S, Coppens M	+	N/A	+	?	?	–	+
Ong S, Young BE	+	N/A	+	?	?	?	+
Peng YD, Meng K	+	N/A	+	?	?	–	+
Piva S, Filippini M	+	?	+	+	N/A	?	+
Poissy J, Goutay J	?	N/A	+	?	N/A	?	+
Simonet A, Chetboun M	+	N/A	+	+	N/A	?	+
Spieza L, Boscolo A	?	N/A	+	+	N/A	?	?
Wu J, Li W	+	N/A	+	?	?	–	+

Results of the modified GUIPS tool. By study participation the article was considered as carrying low risk of bias, if the diagnosis of COVID-19 was clearly stated, unclear risk of bias was given in the case of lacking description and high risk of bias was assessed if suspected or unclear cases were also involved. Study attrition was only assessed in the cases of prospective studies, where green indicates the clear description of follow-up, while yellow means a lacking description. Obesity as the only investigated prognostic factor was considered carrying low risk of bias on individual study level if BMI was assessed. A clear description of the outcomes was needed to achieve low risk of bias. In the case of confounding factors, green indicates a multivariate analysis, yellow means a lacking description and red is associated with the presence of a major confounding factor (e.g. age, gender, treatment etc.). As none of the studies has previously published protocol with statistical plan, we waived the need for the assessment of the statistical analysis reporting.

– High risk   ? Unclear risk   + Low risk
